# How do Chinese and Egyptian science textbooks differ? A cross-country comparative research

**DOI:** 10.1016/j.heliyon.2024.e32380

**Published:** 2024-06-05

**Authors:** Ahmed Hosny Saleh Metwally, Ahmed Tlili, Yiping Wang, Zhimin Li, Jialu Zhao, Boulus Shehata, Dong Yang, Ronghuai Huang

**Affiliations:** a Smart Learning Institute, Beijing Normal University, Beijing, China; b Educational Technology Department, Faculty of Education, Helwan University, Cairo, Egypt; c Department of Education, University of California, Los Angeles (UCLA), Los Angeles, USA; d College of Education for the Future, Beijing Normal University, Zhuhai, China

**Keywords:** Textbooks comparison, Structure analysis, Content analysis, Cognitive expectations, Science textbook

## Abstract

Textbooks have a crucial role in shaping students' knowledge, behaviors, and attitudes in different school subjects. This study compares the structure and content of science textbooks of grade nine in Egypt and China to reveal the common and different features in the textbook design. It opts for a horizontal analysis of four science textbooks in the associated countries. The results revealed that the distribution of science subjects has partial similarities to some extent among the preparatory stage between the Chinese and Egyptian science textbooks besides the overlapping in the associated topics, presenting Biology as a common subject of interest. Moreover, the number of activities distributed within units and subjects have the highest shares in the Chinese textbooks, and most of the activities in the Egyptian textbooks focused on Chemistry and Physics subjects. In addition to the structure analysis, this study also explored the textbooks content in both countries, covering three dimensions: (1) cognitive expectations, (2) learning goals, and (3) efficiency of illustration. The results provide valuable insights for textbook designers and curriculum developers to enhance the quality of science curricula and textbooks. Therefore, the study recommends considering instructional design and lesson plans when distributing the learning activities and developing international standards for designing school science textbooks.

## 1 Introduction

Textbooks play a crucial role in shaping education and enhancing learning outcomes as they have been developed to facilitate pedagogical practices [1]. They can be used as a significant source of knowledge for teaching specific subjects [2], and an objective communication medium, which allows students to obtain correct information and protects the right to education [3]. Teachers often use textbooks to develop their curriculums to support students [4]. The analysis of textbooks therefore can support researchers in understanding the effectiveness of particular schemes and methods to reveal the requirements for teaching and curriculum development [5]. Several studies, in this context, pointed out the importance of analyzing students' textbooks in order to understand their achievements in a given subject [[6], [7], [8], [9]].

There is a significant interest in science textbook research. Vojíř and Rusek [10] provided a comprehensive mapping of science education textbook research trends through a systematic literature review. They pointed out that there is a growing research interest in science textbooks with considerable research in Europe and the USA. Khine [2] provided a critical analysis of science textbooks by evaluating instructional effectiveness through various aspects, highlighting the quality evaluation of textbooks and several analytical instruments (e.g., survey questionnaires, rubrics, grids, criteria, ethnographic content, and reflexive document analyses, etc.), language analysis, and content analysis of textbooks.

### 1.1 Importance of comparing textbooks

Comparative studies of textbooks in different countries are crucial. On the one hand, textbooks are important learning resources that enhance student ability and achievement [11]. A comparison of textbooks helps to understand the possible reasons for the differences in performance of students in different countries [[12], [13], [14]]. This is of great importance in today's world since international large scale assessment, such as PISA (Programme for International Student Assessment) and TIMSS (Trends in International Mathematics and Science Study), has changed the landscape of global education, where education has been reimagined and reshaped on a global scale based on these instruments instead of being constrained to the domestic standards. For example, by analyzing Korean and American textbooks, Hong and Choi [7] found that although American students learn certain topics later than Korean students, American textbooks provide students with more opportunities to explain, reason, and solve problems using multiple representations.

On the other hand, due to the different cultural backgrounds and epistemologies, different countries have their own unique education systems, which lead to different teaching materials and teaching designs [15]. Kang and Kilpatrick [16] highlighted that curricula or textbooks are designed from a didactic transposition under the influence of a given country's various historical and cultural dimensions. Building on this perspective, cross-country textbook analysis can help identify the different knowledge and information that each country teaches, as well as understand the education system and teaching methods in those countries [[17], [18], [19], [20]]. In the same vein, Howson [21] revealed that international comparative studies of textbooks can help to learn a lot about country differences. For example, Takeuchi and Shinno [22] found that, in terms of symmetry and transformations presented in lower secondary mathematics textbooks, the textbooks from England exhibit stronger connections with real-life situations and practical applications. In contrast, the textbooks from Japan place greater emphasis on geometric proofs. In a textbook comparison study examining the developmental course of the fraction concept, Yang et al. [23] asserted that textbooks from the United States, in comparison to those from Singapore and Taiwan, are more inclined to provide clear explanations of problems within real-life contexts, which is highly recommended by professional organizations.

From a teaching perspective, analyzing textbooks across countries can help in explaining the differences in teaching practices. Therefore, a textbook analysis can reveal insights into the process of teaching and learning for a given country [24]. Textbook analysis can also serve as an avenue for investigating curricula reforms [8,25,26].

Beyond the education perspective, the analysis of textbooks across countries can help in understanding the different societal values, including gender bias and equality, that a given country is promoting through its curricula and textbooks [27].

Based on the aforementioned discussion, analyzing and comparing textbooks in various countries can support recognizing the similarities and differences between educational systems in these countries [28], hence contributing to the improvement of textbooks design, as well as the associated teaching and learning practices. Consequently, textbook analysis and comparison (national or international, etc.) is considered one of the crucial directions in the field of textbook research [22].

### 1.2 Importance of comparing science textbooks

Scientific development is one of the most important influencing factors for countries to enter the mainstream of contemporary technology and business. Science education is an important carrier for the modernization and all-around development of various countries and the development of human resources, and it is one of the most important areas in the curriculum. The UNESCO General Conference Report in 1992, cited by Lewis and Kelly [29], suggested that science and technology related education plays an important role in improving cultural and material conditions of people.

Some research studies focused on science textbook analysis within a single country, such as Canada, Sweden, and China [[30], [31], [32]]. Five editions of biology textbooks in China were analyzed to examine the alignment between curriculum standards and textbooks, using content areas and cognitive levels [33]. The study's findings revealed the unbalanced distribution of curriculum standards and textbooks among different core concepts and cognitive levels besides the separation between textbooks and curriculum standards. Li et al. [34] further assessed integration of the nature of science (NOS) into some high school biology textbooks in China. In addition, senior high school physics textbooks in Indonesia were analyzed based on inquiry practices [35]. The findings explored that in these textbooks, physics topics were more cognitively oriented than epistemic or socioculturally oriented.

There are also studies that concentrated on the comparison and analysis of textbooks between various countries. For instance, Erbas et al. [36] compared the mathematics textbook in United States, Singapore, and Turkey. Wang et al. [37] compared textbooks between England and Shanghai. Stadermann et al. [38] analyzed physics textbooks of 15 European countries and revealed that several topics are commonly taught across the countries, while there are differences in terms of the designed learning activities and teaching practices. An and Chua [39] conducted an analysis of Chemistry textbooks between China and Malaysia and found that there is a difference between the textbooks in the sequence in which the chemistry concepts and their application to nature were introduced. Furthermore, Akpınarlı et al. [40] conducted an exploratory comparison between Türkiye and Germany in biology textbooks.

While most of the research in the literature focused on comparing Western and non-Western science textbooks, less attention has been paid to comparing two non-western countries' science textbooks. In this context, Wang et al. [27] compared Chinese and Egyptian science textbooks, but they only focused on gender representation instead of the learning content. This denotes that science textbook comparison usually has different scopes. For instance, Li et al. [32] and Ma et al. [41] studied scientific inquiry activities in science textbooks, whereas Hong and Choi [42] focused on how liner functions are presented in different countries' textbooks. Moreover, from the perspective of language, there is a difference in which science textbook authors utilize linguistic elements to convey scientific knowledge, which indicates interpretable differences through various fields of science, including physics, biology, chemistry, and other related disciplines [43]. Therefore, comparing science textbooks in different countries can reveal the science competencies and knowledge that different countries value [20,44].

## 2 Research gap and study objectives

This study aims to address the knowledge gap related to the current topic from two perspectives: (1) the major focus on science education analysis neglected the Asian and Arab regions; and (2) when comparing textbooks, most research did not pay much focus on learning expectations which can reveal what type of science knowledge and outcome an educational system is valuing. Each perspective is further elaborated below.

### 2.1 Region-focused analysis of science education materials

There are large differences in the amount of textbook research in different countries. Of the research papers focusing on textbook analysis published in journals indexed by the Web of Science between 2000 and 2018, the majority of published papers were on European and North American textbooks. Of these, more than half of the European countries were identified [10]. Most of the comparative studies on science education neglected Asia and the Arab region.

Advances in the field of science and technology are a key goal in many Arab countries [45], as the prosperity of science and technology is considered essential for economic and social development [46]. Particularly, Egypt has recognized the significance of science as a crucial discipline to promote and enhance the understanding of quality learning [47]. Starting from the 1960s, Egypt has provided considerable support to students at primary, preparatory, and secondary levels by considering science as a fundamental subject in their education [47]. In 2003, the Egyptian Ministry of Education (MOE) released the National Standards for Education in Egypt (NSEE) as a national project and placed a strong emphasis on highlighting the interconnectedness of science, technology, and society from three distinct dimensions: (a) science and technology; (b) science from a social and individual perspective; (c) the history and nature of science.

China also considers science education and the development of students' scientific literacy as primary objectives of science education reform [48]. Since 2001, China has implemented a new round of basic education curriculum reform, with a specific focus on science education for grades 7–9. The aim is to provide students with a deep understanding of scientific knowledge, proficiency in scientific processes and methods, and comprehension of the nature of science. This reform intends to cultivate students' abilities in innovation, critical thinking, and practical application of scientific concepts [49]. Accordingly, China undertook a revision of the science curriculum standards in 2011 to advance science education.

Motivated by a prior research effort that sought to compare science textbooks in China and Egypt from a social semiotics perspective [27], this study extends the research endeavors to further reveal other faces of the science textbooks comparison in the associate countries. Considering that education is shaped by cultural value within a society [50], it would be interesting to expand the scope of science textbooks comparison between China and Egypt. No study, to the best of our knowledge, conducted cross-country comparative research focusing on Chinese and Egyptian science textbooks. Thus, the present study will extend the literature from this perspective.

### 2.2 Lack of a comprehensive analysis of science textbooks

In most of the current research on textbooks, the extant studies focused on the content of textbooks [36,42,51,52], visual design [36,53]. Research on textbooks primarily focuses on three main areas related to the content, its conception, and presentation of the learning content [10]. There are limited studies on metrics, such as structure [54], questions [42], and learning expectations [55,56]. These metrics, such as learning expectations, could help to understand which type of knowledge a given country aims to emphasize when teaching science. This could help to further understand the obtained students' learning achievements later on. Moreover, noticeable research on textbooks comparison sought to analyze the textbooks' scientific content or activities, for example, the scientific literacy in Egyptian textbooks [47]; data activities and instructional supports [57]; scientific inquiry activities of Chinese science textbooks in high schools [41], overlooking the holistic comparison of structure and content of preparatory science textbooks. Therefore, this present study goes beyond the single-element analysis within the textbook structure (i.e., analysis of images or activities, etc.) to comprehensively analyze the textbook learning content. Specifically, all textbook elements were analyzed to identify the different types of cognitive expectations and learning goals, hence providing more in-depth on the similarities and differences between Chinese and Egyptian science textbooks. Therefore, the analysis, as depicted in Fig. 1, covers two main parts, namely: (1) textbook structure of grade 9 that involved the structural characteristics, such as counts of units and lessons, illustration counts, distribution of the subjects, distribution of the activities, units, and lessons, and units structure; (2) textbooks content of grade 9 with cognitive expectations, learning goals and efficiency of illustrations.

**Fig. 1:**
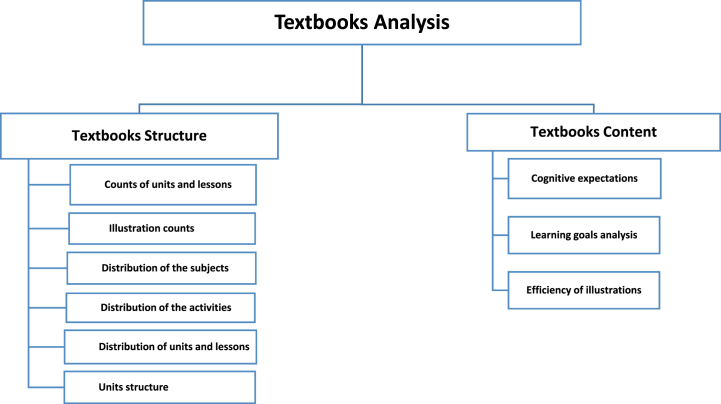
Analysis scheme of the science textbooks.

**Fig. 2:**
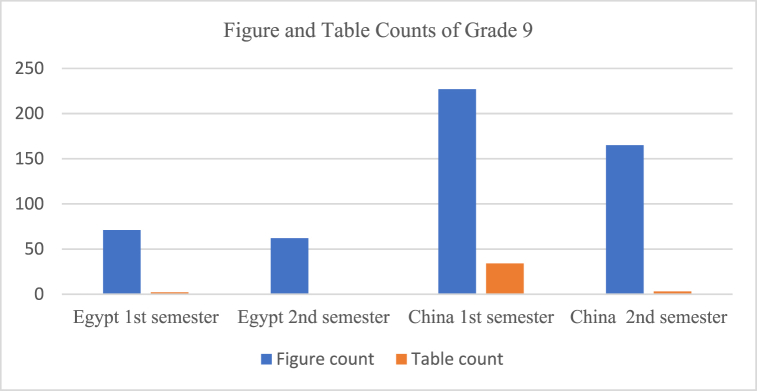
Figures and tables counts.

To sum up, this study conducts a comprehensive cross-country comparative analysis of Chinese and Egyptian science textbooks to explore the degree to which these countries have similar or different science textbooks. Particularly, ninth-grade science textbooks were chosen because the content of ninth-grade textbooks is more sophisticated and challenging as it aims to foster students' abilities in scientific thinking and encourage them to engage in a deep analysis of concepts building upon the knowledge and skills acquired by students in the previous years, specifically grades seven and eight of the preparatory stage [27,58]. Additionally, according to Piaget's theory, this age represents the fourth stage of cognitive development, formally denoted as the operational stage 12–15 years [59]. Accordingly, this present study answers the following research questions:

RQ1 To what degree does the structure of Chinese and Egyptian ninth-grade textbooks vary in terms of units, lessons, illustration counts, distribution of the subjects, activities, and unit structure?

RQ2 To what degree does the content of Chinese and Egyptian ninth-grade textbooks vary in terms of cognitive expectations, learning goals, and efficiency of illustrations?

This study contributes to the literature of science textbook research by revealing and recognizing the similarities and differences aspects of science textbooks in Egypt and China. This helps to identify the current state of the science textbooks in these countries (and maybe regionally), hence enhancing the design and development phases to be reflected in the quality of school textbooks in the future.

## 3 Method

### 3.1 Materials

Four textbooks, two from Egypt and two from China, all in Grade 9 were selected for this study. The selection process was guided by the text comparison framework developed by Huang et al. [60]. Egypt and China have different systems of textbook publication. In Egypt, science textbooks are approved by the Ministry of Education and Technical Education of Egypt and the same textbooks are used by all school students in the country. In China, under the curriculum standard designed by the Ministry of Education, different publishers take responsibilities for textbook publications such as People's Education Press and Zhejiang Education Press. Different science textbooks are therefore used by different regions. This study chose the textbooks published by Zhejiang Education Press for analysis. This particular publisher was chosen because of the similarity of its science textbook structure to that of the Egyptian textbooks, i.e., a separate textbook is available for each semester and includes content from different subjects like physics, chemistry and biology [27]. Additionally, since 1990, the Zhejiang Education Press played a significant role in the development of science textbooks that align with the science curriculum reform initiated in the province of Zhejiang, one of the most developed areas in China, both culturally, technologically, and economically [61]. In addition, science textbooks from Zhejiang Education Press have also been studied by many scholars [27,62].

### 3.2 Coding scheme

Based on the textbook analysis scheme, we coded structure and content guided by the comprehensive framework of comparison provided by Huang et al. [60]. The textbook structure includes (1) counts of units and lessons, (2) illustration counts, (3) distribution of the subjects, (4) distribution of the activities, (5) distribution of units and lessons, and (6) unit structure. The textbook content covers three dimensions: (1) cognitive expectations, (2) learning goals, and (3) efficiency of illustration.

As suggested by Kar et al. [63], the cognitive expectation of each given assignment/problem with the textbook was coded into six categories, namely: procedural knowledge, conceptual knowledge, representation, problem-solving, reasoning, and problem-posing, as shown in Table 1. Procedural knowledge generally involves algorithms or operations. Procedural knowledge is also related to the operation of scientific experimentation. Conceptual knowledge often requires students to recall and explain concepts. Representation expects students to use various formats (text, tables, pictures, etc.) to answer the question. Problem-solving is commonly tightly integrated into everyday life and students are asked to solve a problem based in the real-world setting. Reasoning requires students to explain their thinking process. Problem posing mostly requires students to ask or revise the question according to the specific situation.

**Table 1:** Analysis scheme of the cognitive expectations.

Dimension	Category
Cognitive expectation	Procedural knowledge (PK)
	Conceptual knowledge (CK)
	Representation (RP)
	Problem-solving (PS)
	Reasoning (RS)
	Problem posing (PP)

It is worth noting that a problem might be coded into more than one category. For example, an assignment might belong to problem-posing, problem-solving, and reasoning. Table 2 presents examples of assignments in the textbooks and their codes.

**Table 2:** Sample problems in the textbooks and their coding.

Example	Cognitive expectation
1. How many seconds does a day has?	PK
2. One of the measurement units of speed is … or ….	CK
3. Write down the name of the chemical instrument shown in the picture below.	RP
4. Tie a small iron block to one end of a long rope to make a pendulum (Fig. 1, Fig. 2, Fig. 3, Fig. 4, Fig. 5, Fig. 6, Fig. 7, Fig. 8). To measure the time (period) it takes to swing back and forth, how can it be measured more accurately? Can you make a pendulum with a period of 1s?	PS/RS
5. The following is the incubation time of several bird eggs, please speculate what the influence the incubation time?	RS
6. Complete a survey report on campus (or park, farmland) biology. The content should include time, location, weather, survey route, type, quantity and living environment of creatures seen, experience after survey, etc.	PP/PS/RS

When coding the learning goals in textbooks, they were categorized into six categories according to the complex requirements of students' abilities [60]: (1) understanding basic information/knowledge (BI); (2) understanding complex information/knowledge (CI); (3) theorizing, analysing, and solution exercises (TE); (4) understanding the use of tools, routines, and scientific processes (TP); (5) solving real-world problems (SR) [17]; and (6) acquiring values and attitudes (AA). Table 3 shows the analysis scheme of the learning goals and sample goals in the textbooks and their coding.

**Table 3:** Analysis scheme of the learning goals with coding and examples.

Dimension	Category	Example
Learning goals analysis	Understanding basic information/knowledge (BI)	After learning this unit, you will be able to distinguish uniform motion and variable motion.
	Understanding complex information/knowledge (CI)	After finishing this section, you will understand how the lens is imaged.
	Theorizing, analyzing, and solving exercises (TE)	Learn to analyze, synthesize, reason, and cultivate scientific research capabilities.
	Understanding the use of tools, routine procedures, and science processes (TP)	Learn to measure the length of the object.
	Solving real-world problems (SR)	Understand the local water and soil conditions.
	Acquiring values and attitudes (AA)	Understand the greatness of the Creator by learning about galaxies and the solar system.

It is worth noting that learning expectations refer to the specific knowledge, skills, and attitudes that students are expected to acquire as a result of their educational experiences. These expectations are often outlined in learning goals and serve as benchmarks for student achievement. On the other hand, cognitive expectations focus on the cognitive processes that students are expected to develop or utilize in their learning and pertain to the mental activities involved in learning, as shown in Table 1. Therefore, cognitive expectations are often tied to how students process information rather than just what they know.

According to Mikk [64], illustrations in textbooks are classified into four categories: representation, organization, interpretation, and transformation. If an illustration simply reflects the same content as the text, it is coded as “representation” (R). If an illustration summarizes or categorizes textual information, it is coded as “organization” (O). If an illustration explains the information and helps the reader understand it, it is coded as “interpretation” (I). If an illustration re-encodes the information in a more memorable form, it is coded as “transformation” (T) [64,65]. Table 4 presents the coding scheme of the efficiency of illustration and sample illustrations in the textbooks and their codes.

**Table 4:** Analysis scheme of the efficiency of illustration with coding and examples.

Dimension	Category	Example
Efficiency of Illustration	Representation (R)	1. Dog's family.
	Organization (O)	2. Take the liquid with a dropper.
	Interpretation (I)	3. Reflection of incident light from concave mirror.
	Transformation (T)	4. Interconversion of the three states of matter transformation.

The Chinese and Egyptian science textbooks were coded by four coders who speak Chinese and Arabic and have good expertise in comparative research and curriculum development. In addition, a translation team has translated the two Egyptian textbooks into Chinese. The coding process took two months, scheduling weekly meetings to discuss and resolve any doubts and conflicts in coding. This ensured the reliability of the coding results. Inter-rater reliability reached 0.95 between the coders, which is considered a very good agreement [66].

## 4 Results

This section presents the study findings to offer overarching answers to the research questions, aiming to reach a better comprehensive analysis of the science textbooks regarding the textbooks' structure and content in Egypt and China.

### 4.1 Textbooks structure

This section focuses on identifying the Chinese and Egyptian textbook structure of Grade nine to offer significant future insights for evaluation and development. Therefore, it presents how different contents are combined and sequenced within the textbooks regarding the following aspects: counts of units and lessons, illustration, distribution of the subjects, distribution of the activities, distribution of units and lessons, and unit structure.

#### 4.1.1 Counts of units and lessons

There are consistent four units in the science textbooks with a variance of lessons. While there are 15 lessons over grade nine in the Egyptian textbooks, 45 lessons exist in the Chinese textbooks. The Chinese textbooks tend to include more lessons compared to the Egyptian textbooks (see Table 5).

**Table 5:** Structure distribution of ninth-grade science textbooks in Egypt and China.

Textbooks of grade 9	Units	Lessons
Egypt 1st semester	4	8
Egypt 2nd semester	4	7
China 1st semester	4	25
China 2nd semester	4	20

#### 4.1.2 Illustration counts

Textbook illustration is one of the important aspects of structure. The Chinese textbooks are found to use a great number of figures, reaching 392 figures in total compared to the Egyptian textbooks which reached 133 figures. On the other hand, while the Chinese textbooks had 37 tables, the Egyptian textbooks had only two tables with a lack of tables in the 2nd semester.

#### 4.1.3 Distribution of the subjects

In Egypt's ninth-grade science textbooks, Physics and Biology subjects are represented as the largest proportion with three units of each subject, while Geography and Chemistry have the lowest representation with one unit for each subject. In Chinese ninth-grade science textbooks, Biology is dominant with four units, followed by Geography and Chemistry with two units equally, and Physics with one unit as presented in Table 6. This distribution is in line with the overall distribution of the subject of the preparatory stage mentioned in the last section.

**Table 6:** Distribution of the subjects in both countries.

	Chemistry	%	Physics	%	Biology	%	Geography	%
Egypt	1	12.5	3	37.5	3	37.5	1	12.5
China	2	22.2	1	11.1	4^a^	44.4	2^a^	22.2

a Note:There is one unit that has both geography and biology content.

#### 4.1.4 Distribution of the activities

Recognizing the activities related to the units and subjects can indicate to what extent these activities are distributed to achieve the learning goals and whether it is sufficient in terms of comparing their quantity in both countries. Regarding the distribution of activities within units, Table 7 shows the total number of activities, where there are 23 activities in the Egyptian science textbooks and 65 activities in the Chinese science textbooks. Most of the activities are included in the first semester's Chinese textbook with 51 activities in total and 32 activities in units 1 and 2, covering Chemistry topics. However, the overall number of the 2nd semester's Egyptian textbook activities overreached its pair in the Chinese textbook. Generally, the distribution of activities within units is not regular, for example, some units in the Egyptian textbooks either do not have activities or have only one activity in the unit.

**Table 7:** Distribution of activities on the unit.

Textbook	Unit 1	Unit 2	Unit 3	Unit 4	Total	
Egypt 1st semester	1 (Physics)	5 (Physics)	1 (Geography)	1 (Biology)	8	23
Egypt 2nd semester	10 (Chemistry)	4 (Physics)	1 (Biology)	0 (Biology)	15	
China 1st semester	17 (Chemistry)	15 (Chemistry)	13 (Physics)	6 (Biology)	51	65
China 2nd semester	3 (Geography/Biology)	3 (Biology)	6 (Biology)	2 (Geography)	14	
Total	31	27	21	9	88	88

**Fig. 3:**
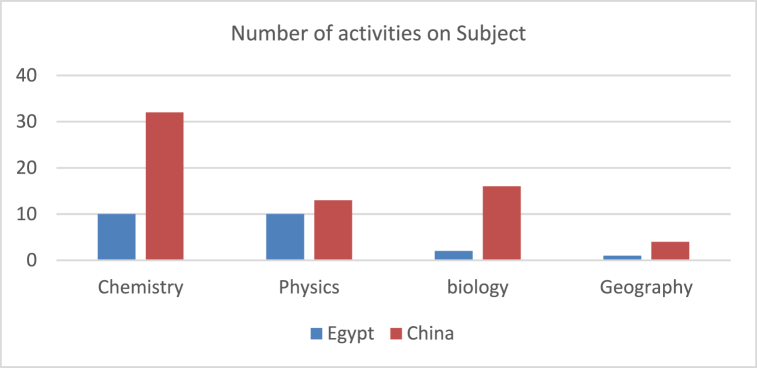
Distribution of activities on the subjects.

In terms of the distribution of activities within subjects, the Chinese textbooks have several activities that reached 32 activities. They covered the Chemistry lessons, followed by Biology and Physics. On the other hand, Egyptian textbooks had 10 activities for the Chemistry and Physics lessons. Although Physics and Biology are the dominant represented subject within units in the Egyptian textbooks, we noticed that most of the activities are planned for Physics and Chemistry subjects. Meanwhile, in the Chinese textbooks, Biology has over four units as the dominant subject, but the number of activities does not reflect this rate with the highest amount of activities. Moreover, there is a scarcity of activities to support Geography lessons in both countries as depicted in Fig. 3.

#### 4.1.5 Distribution of units and lessons

Although science textbooks of the ninth grade in Egypt and China have an equal number of units in total (8 units), the total number of lessons is different over the units and the semester as shown in Table 8. In Egypt, the science textbooks have 15 lessons covering several topics with a minimum of one lesson and a maximum of three lessons per unit (e.g., force and movement, reproduction and continuation of species, chemical reaction). The Chinese textbooks included 45 lessons ranging between four and eight lessons per unit to demonstrate different topics such as “matter and its Changes", "metabolism and balance", and "biology and environment”.

**Table 8:** Units and lessons of 9th-grade textbooks.

Egypt			China		
	Unit	Lesson		Unit	Lesson
**Book 1**	**Force and movement**	1. Linear motion 2. Graphical representation of linear motion 3. Scalar and vector physical quantities	**Book 1**	**Matter and its Changes**	1. Changes in matter 2. The acidity of the substance 3. Common acid 4. Common base 5. Reaction between acid and base 6. Several important salts
	**Light energy**	1. Lens 2. Mirror		**Substance transformation and material utilization**	1. Metallic material 2. Chemical properties of metals 3. Organics and organic synthetic materials 4. Classification of substances 5. Transformation of matter 6. Material utilization and development
	**Universe and solar system**	1. Universe and solar system		**Transformation and conservation of energy**	1. Energy and its forms 2. Mechanical energy 3. Measure of energy conversion 4. Simple machinery 5. internal energy of an object 6. Electrical energy 7. Nuclear energy 8. Conversion and conservation of energy
	**Reproduction and continuation of species**	1. Cell division 2. Asexual reproduction and sexual reproduction		**Metabolism and balance**	1. Food and nutrition 2. Digestion and absorption of food 3. Transport of substances in the body 4. Gain of energy 5. Homeostasis of substances in the body
**Book 2**	**Chemical reaction**	1. Chemical reaction 2. Chemical reaction rate	**Book 2**	**Evolved nature**	1. Human understanding of the universe 2. The formation of the solar system and the evolution of stars 3. The evolution of the earth and the origin of life 4. Biological evolution 5. Genetics and evolution
	**Physical properties of electrical energy and radioactivity**	1. Physical properties of electric current 2. Current and electrode 3. Radioactivity and nuclear energy		**Biology and environment**	1. Interrelationship between organisms and the environment 2. Population 3. Biomes 4. The structure and function of ecosystems 5. Ecosystem stability
	**Genes and inheritance**	1. Fundamentals of genetics		**Human health**	1. Health 2. Threats from microbes 3. Body defense 4. Non-communicable diseases 5. Human movement system and healthcare 6. Healthy lifestyle
	**Hormones**	1. Hormone regulation in the human body		**Sustainable development**	1. Human development and environmental issues 2. Energy and its utilization 3. Low carbon life 4. Achieve sustainable development
**Total**	**8**	**15**		**8**	**45**

#### 4.1.6 Unit structure

**Fig. 4:**
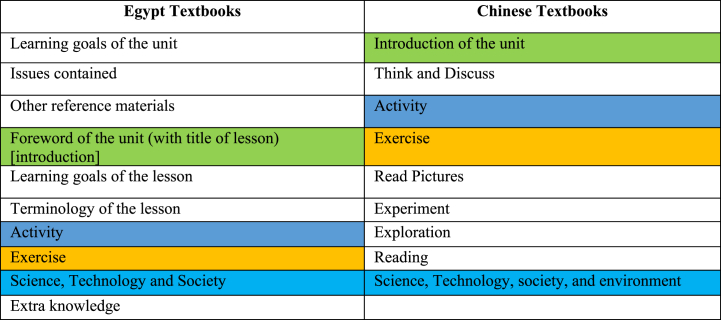
Unit structure.

We sought to identify the unit structure of the textbooks and how far the organization and the included components are similar or different in the units. Therefore, we extracted the main components of the units in the same order of organization in the textbook. We then highlighted the similar components in the Egyptian and Chinese textbooks as shown in Fig. 4. While there are a few common components, such as *introduction*, *activity*, *exercise*, *and science*, *technology and society*, there are some components that do not match (e.g., *learning goals*, *issues contained*, *other reference materials*, *terminology of the lesson*, *extra knowledge*, *think and discuss*, *reading*, and *exploration*), which denote lacking a standard to organize the unit structure. Surprisingly, the Chinese textbooks lacked approaching the learning goals and tended to concentrate on the discovery activities, unlike the goal-oriented textbooks in Egypt that listed the learning goals at the beginning of the unit and lessons besides its propensity to supply the unit with extra resources and knowledge.

### 4.2 Textbooks content

This section presents how various contents are presented in the Chinese and Egyptian science textbooks on the aspects related to cognitive expectation, learning goals, and efficiency of illustration.

#### 4.2.1 Cognitive expectations

##### 4.2.1.1 Type of cognitive expectations

**Fig. 5:**
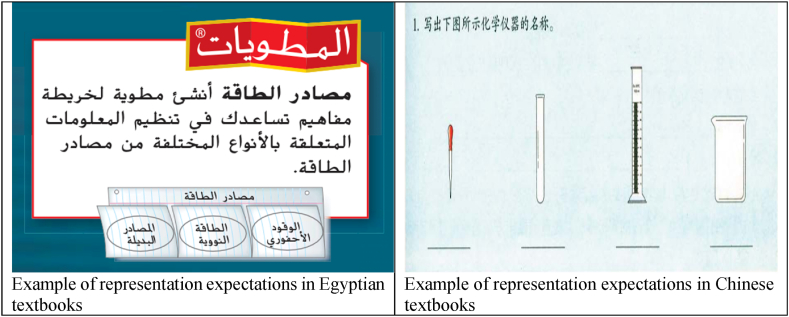
Sample of cognitive expectations.

We analyzed the cognitive requirements that enable students to use knowledge and skills to solve exercises. It can be divided into six levels: procedural knowledge, conceptual knowledge, representation, problem-solving, reasoning, and problem posing. The six cognitive requirements are in hierarchical order. Procedural knowledge, conceptual knowledge, and representation are fundamental cognitive requirements since they are related to the comprehension of basic concepts. Problem-solving and reasoning are more advanced capacities compared to the first three categories, which not only require students to understand the concepts and the connections between concepts but also how these concepts are applied in a real-world setting. Students need to understand the scientific mechanism to fulfill these two cognitive requirements. Problem posing is a further step than problem solving and reasoning. Problem posing means students think in the way scientists think about science, which is a creative and innovative way of thinking. Results suggest that there is a considerable disparity in the cognitive expectations between the countries. Apparently, most of the exercises in the Chinese and Egyptian textbooks covered the representation level, which required solutions in the form of pictures, tables, or interpretations, as shown in Fig. 5.

**Fig. 6:**
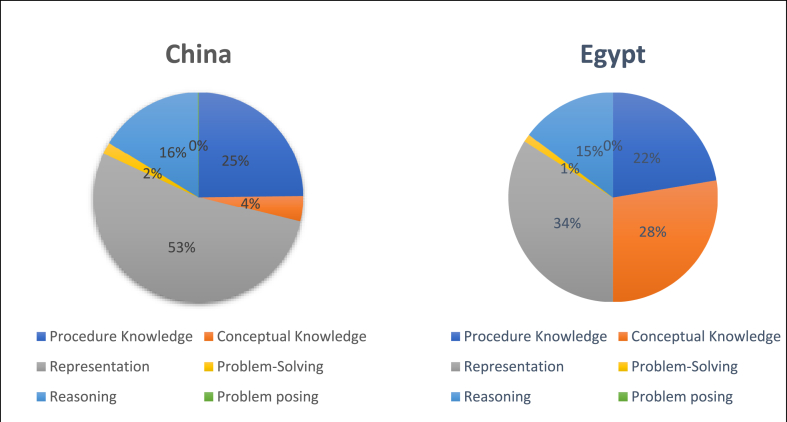
Comparison of cognitive expectations.

As shown in Fig. 6, together, the basic cognitive requirement, such as Procedure knowledge, Conceptual knowledge, and Representation, accounts for more than 80 % of the total cognitive requirement in both Chinese and Egyptian textbooks. The more advanced cognitive requirements including problem-solving and reasoning together account for more than 15 % in both countries. Both countries have very limited problem-posing requirements for the students within their science textbooks. It can be argued that both countries aim to enhance students' basic cognitive requirements but have insufficient requirements for more advanced cognitive skills.

There is a disparity between the two countries in terms of the basic cognitive requirement, Chinese science textbooks emphasize representation (53 %) much more than procedure knowledge (25 %) and conceptual knowledge (4 %). Egyptian science textbooks have a similar amount of the three categories: representation (34 %), procedural knowledge (22 %), and conceptual knowledge (28 %). It can be argued that Egyptian textbooks highlight the importance of the comprehension of basic concepts, which is more static, whereas Chinese textbooks provide more opportunities for students to exercise the concepts by applying them through pictures, tables, or interpretations in a more interactive way.

**Fig. 7:**
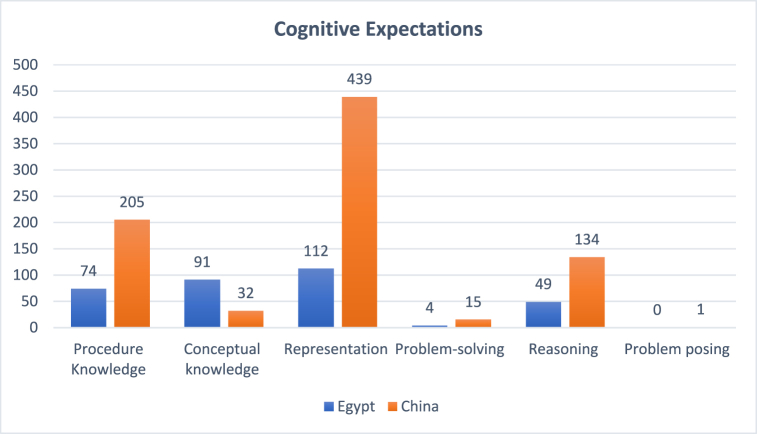
Counts of cognitive expectations in the textbooks.

As for more advanced cognitive requirements, the Chinese science textbooks have slightly more cognitive requirements than the Egyptian science textbooks. In the Chinese textbooks, 16 % of the cognitive requirement is related to reasoning, while only 2 % is related to problem-solving. Similarly, in the Egyptian textbooks, 15 % of the cognitive requirement is related to reasoning, while only 1 % is related to problem-solving. It can be argued that both Chinese and Egyptian science textbooks emphasize scientific knowledge thinking in an abstract way that does not have much connection to the real-world setting. It is worth noting that despite the proportionality, Chinese and Egyptian science textbooks have a close amount of basic and advanced cognitive expectations. In terms of the absolute number, the Chinese textbook's cognitive expectation exceeded the Egyptian textbook, as shown in Fig. 7.

##### 4.2.1.2 Cognitive expectations and cognitive activities

**Fig. 8:**
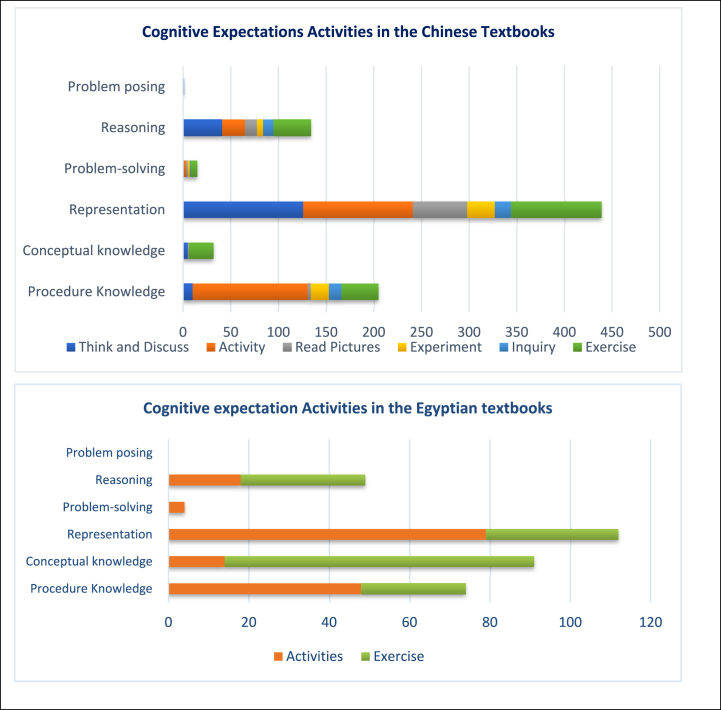
Cognitive expectation activities.

Cognitive expectations can be represented in the science textbooks through several types of cognitive activities. The science textbooks in China are supplied with various types of tasks, including *Think and Discuss, Activity, Read Pictures, Experiment, Inquiry,* and *Exercise*. Among *representation* as the dominant level in the Chinese textbooks, *Think and Discuss, Activity, and Exercise* are the most prominent expectations to achieve the learning goals, whilst *Activity* is often used in the procedure knowledge level, with less representation of *Inquiry* as depicted in Fig. 8. Meanwhile, the cognitive tasks in Egypt's textbooks involve *Activities* and *Exercises*, unlike the Chinese textbooks, leveraging *Activities* for the representation and procedure knowledge levels and *Exercises* for the conceptual knowledge level.

##### 4.2.1.3 Cognitive expectations by subjects

In relation to the cognitive expectation levels, we analyzed the distribution of cognitive expectation by subjects. As presented in Table 9, nearly half of the cognitive expectations are associated with Physics, followed by Chemistry, and Biology in the Egyptian textbooks, whilst these activities are distributed on Biology, Chemistry, and Physics respectively with different proportions in the Chinese textbooks. Hence, a small percentage of cognitive activities in Geography (13 % in Egypt and 8.5 % in China) were included in the textbook.

**Table 9:** Distribution of cognitive expectation on subjects.

Subjects	Cognitive Expectation Activities			
	Egypt	%	China	%
Physics	158	48	161	19.5
Chemistry	95	29	279	34
Biology	64	19	317	38
Geography	13	4	69	8.5

**Fig. 9:**
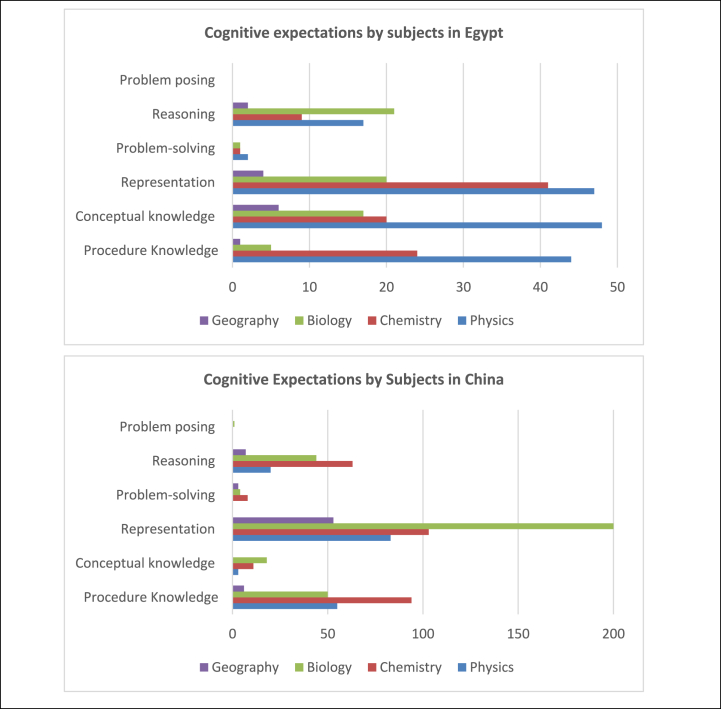
Distribution of cognitive expectation levels on subjects.

Fig. 9 shows that Physics has the highest share of both procedure knowledge, conceptual knowledge, and presentation levels, followed by Chemistry on the same cognitive levels in the Egyptian science textbooks. In the Chinese science textbooks, there is a tendency to include representation activities with Biology topics and considerable tasks focused on procedure knowledge, representation, and reasoning with Chemistry.

#### 4.2.2 Learning goals analysis

**Fig. 10:**
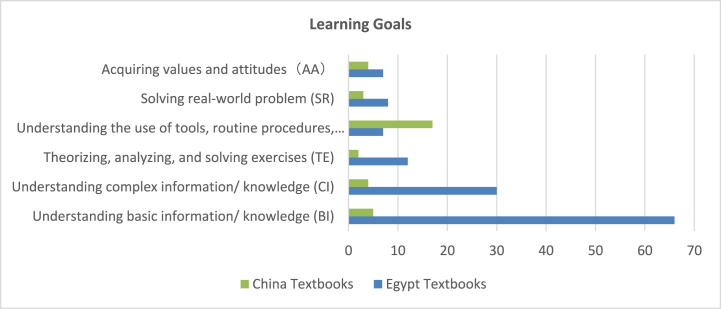
Distribution of learning goals.

**Fig. 11:**
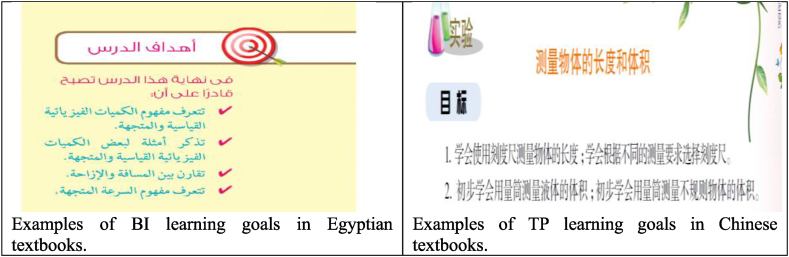
Samples of learning goals levels.

The learning goals in the science textbooks were analyzed based on six levels: (1) understanding basic information/knowledge (BI); (2) understanding complex information/knowledge (CI); (3) theorizing, analyzing, and solving exercises (TE); (4) understanding the use of tools, routine procedures, and science processes (TP); (5) solving real-world problems (SR); and (6) acquiring values and attitudes (AA). 130 learning goals were identified in the Egyptian science textbooks, while 35 learning goals were identified in the Chinese science textbooks. Nearly half of the overall learning goals in the Egyptian textbooks (51 %) looked at helping students to understand basic information and knowledge and 25 % of the goals listed for understanding complex information. On the other hand, 49 % of the learning goals in the Chinese textbooks aimed to support students for understanding the use of tools, routine procedures, and science processes (see Fig. 10, Fig. 11). This indicates that the variation across countries regarding the distribution of learning goals levels is not balanced to reflect preferences for covering specific goals. It is worth noting that although the science subjects are mainly intended knowledge and practical skills, we figured out including learning goals related to acquiring values and attitudes through learning.

#### 4.2.3 Efficiency of illustrations

**Fig. 12:**
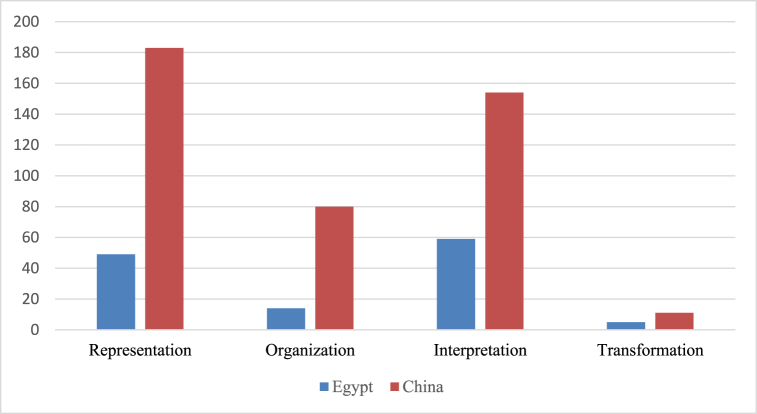
Counts of illustration dimensions.

The efficiency of illustrations is represented according to the following dimensions: representation, organization, interpretation, and transformation. The illustrations in the Chinese science textbooks are predominated on all dimensions as depicted in Fig. 12.

**Fig. 13:**
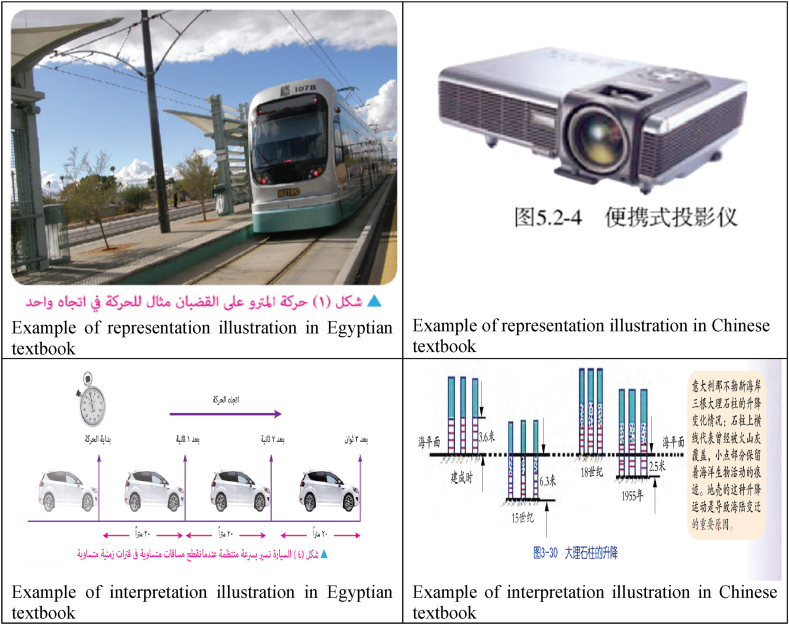
Samples of efficiency of illustration analysis.

Generally, most illustrations in both Egyptian and Chinese textbooks are functioned as representation and interpretation. The number of related figures to the “representation” dimension demonstrates a very low amount of information and “interpretation” that explain difficult concepts, surpassing 150 figures in the Chinese textbooks for each dimension, while the number of the “interpretation” dimension of the Egyptian textbook reached 59 figures. In the Chinese textbook, ‘representation’ accounts for 43 %, followed by ‘interpretation’, which accounts for 36 %. ‘Organization’ accounts for 19 % and ‘transformation’ accounts for only 3 %. In the Egyptian textbook, ‘interpretation’ accounts for 46 %, followed by ‘representation’ (39 %), ‘Organization’ accounts for 11 %, and ‘transformation’ accounts for only 4 %. Overall, there is a lack of “transformation” figures that encompass an enormous amount of information in both countries, as shown in Fig. 13.

## 5 Discussion

### 5.1 Structure analysis

This study conducted a cross-country comparison of ninth-grade science textbooks regarding their structure and contents. Although the Chinese and Egyptian science textbooks have the exact number of units (four units each), the Chinese textbooks include a higher number of lessons (45 lessons) while the textbooks in Egypt cover 15 lessons. This raises the question of whether or not the quantity of lessons influences achieving the learning goals effectively. Overall, the count of lessons, figures and tables in the Chinese science textbooks overreached the Egyptian textbooks.

There is a clear overlap in the associated science topics among the science textbooks in Egypt and China. This indicates the significance of teaching basic topics in the preparatory stages for Egyptian and Chinese students with various degrees of coverage over the units. Distribution of subjects' analysis encourages developing contents and knowledge maps over the grades to assure fair coverage and avoid unnecessary duplication or overlapping. The findings identified Biology as a common subject of interest in both countries in 9th grade. Particularly, the science textbooks in Egypt pay great attention to Physics and Biology with equal representation and less coverage of Geography and Chemistry, whilst the Chinese textbooks leverage Biology with higher inclusions of topics and a lower scope of Physics content. This reveals changes in discipline tendency in the Chinese textbooks, where physical science was regarded over life science and earth science discipline through three decades [62]. Moreover, this could indicate a fair subject's distribution but does not interpret the higher number of lessons, figures and tables in the Chinese textbooks. It is questionable whether the distribution of subjects could have a relation and reflect on the number of lessons, activities, pages, figures and tables. Therefore, it can be observed from the analysis that the first semester of the Chinese textbook has the peak of lessons, figures and tables with a distribution of two units of Chemistry and a unit of Physics and Biology each. This denotes that Chemistry as a subject requires conducting several steps and following procedures of experiments, which could explain inserting several lessons and illustrations. This is aligned with addressing the design of scientific inquiry activities in the newly published Chinese textbooks [41]. On the other side, the science textbooks in Egypt have two units of Physics in the first semester and two units of Biology in the second semester. However, the counts of lessons and illustrations are relatively near in both semesters.

Furthermore, the number of activities within units and subjects in accordance with the finding reveals the highest shares in the Chinese textbooks. In particular, the first semester of the Chinese textbook contained numerous activities and most of them are identified in Chemistry, followed by Physics, which emphasizes the dominant counts of the first semester of the Chinese textbook. Similarly, most of the activities in the Egyptian textbooks focused on Chemistry and Physics subjects as required planning a substantial set of activities. Furthermore, there is a dearth number of activities in Biology topics in Egypt compared to China in the same subject. Therefore, we emphasize considering the effective planning of activities that represent the relative weight of content and achieve the learning goals, scientific concepts and skills.

Regarding the units' organization of the textbooks, we revealed a few matches of the unit construction between the Egyptian and Chinese science textbooks sections (e.g., introduction, activity, exercise, science, technology, and society). Moreover, the Egyptian textbooks seem to be learning goals-oriented, while the Chinese textbooks opt for exploration activities, scientific inquiry activities, and hands-on experiments. This aligns with China's science course objectives for the preparatory stages that focuses on delivering scientific knowledge through inquiries and scientific processes [67]. This promotes developing standards and criteria to design and evaluate the structure of science textbooks based on existing essential features. Thus, we suggest evaluating the effect of the unit's structural elements on the cognitive load and enhancing learning outcomes for future research. One of the interesting findings in the Chinese science textbooks is that the preparatory stage supports students with the introduction of the unit as scaffolding before studying the science subjects, whereas this introduction appears after “Learning goals of the unit”, “Issues contained”, “Other reference materials” as “Foreword of the unit” in the Egyptian textbooks. This supports the progressive organization of science subjects and supplies students' perception with preliminary knowledge for their learning. Thus, we suggest employing some scaffolding strategies like questioning, modeling, instructing, feeding back, and brainstorming to be used within the introductory units to facilitate students' metacognitive skills [68,69].

### 5.2 Content analysis

In terms of the cognitive expectations of textbooks, both Chinese and Egyptian textbooks have a strong emphasis on the representation type over the cognitive expectations.

Representation is associated with obtaining as well as conveying information efficiently in a scientific investigation. Representation not only requires students to obtain information from the various existing forms other than text, such as tables and figures but also requires students to express their reasoning process through drawing a picture or completing some information in a table, or even observing the experiment. Representation is important because the graph and tables generally contain a density of information, as the proverb suggests: a picture is worth a thousand words. Researchers have also suggested that illustration has significant importance in supporting cognitive processes [70]. It can be argued that representation can facilitate students' skills in understanding, organizing and presenting the data in scientific inquiries, which is one of the fundamental parts of science literacy. In addition to the representation, both Chinese and Egyptian science textbooks highlight the importance of procedure knowledge. Procedure knowledge generally involves algorithms or operations. To be more specific, students are expected to utilize their mathematical knowledge to solve scientific problems. In addition, procedure knowledge is also associated with the comprehension of steps in scientific experimentation. Procedure knowledge is an important prerequisite for students to develop more advanced capacities such as designing scientific inquiry. Therefore, it can be said that both Chinese and Egyptian textbooks emphasize building the basic cognitive capacity of students.

The major difference between the Chinese and Egyptian science textbooks is that Egyptian textbooks emphasize much more conceptual knowledge than the Chinese textbooks. Conceptual knowledge often requires students to recall and explain concepts, which is the building block of scientific reasoning. Nevertheless, the over-emphasizing on the basic concept might lead to ignoring the development of more advanced cognitive skills such as reasoning, problem-solving and problem-posing.

Building on the previous cognitive skills, such as procedural knowledge, conceptual knowledge, and representation, reasoning enables students to explain phenomena scientifically. Yet compared with basic cognitive requirements mentioned in the earlier paragraph, reasoning accounts for a lesser amount in both Chinese (16 %) and Egyptian (15 %) textbooks.

Furthermore, procedure knowledge and reasoning expectation types in the Chinese science textbooks are prominent in Chemistry, where students are asked to conduct experiments following steps or procedures, give explanations, state the reason, or make speculation, while representation activities are related to Biology. Thus, this is observed with a considerable number of activities in Chemistry, the highest share of cognitive activities tasks in Biology, and the wide distribution of Biology subjects on the units that reflected on dominating the procedure knowledge and reasoning expectation types. Based on this finding, the textbooks can further consider in their design incorporating reasoning activities with data, allowing opportunities to learn how to record, analyze, and interpret data [57]. Moreover, the inquiry cognitive tasks were less presented among the cognitive expectations; rather than being simple and not authentic to meet the requirements of scientific inquiry [32]. This is constant with the study of Yang et al. [71] who found that inquiry-based tasks in the Chinese high school biology textbooks do not reflect an appropriate understanding of scientific inquiry. This is also in line withMa et al. [41] who emphasized the lack of scientific inquiry activities in the Chinese textbooks, calling for re-checking of inquiry-based tasks in textbooks. In Egypt, the prevalent cognitive tasks belong to Physics followed by Chemistry and this is in accordance with the distribution of the activities within the subjects and partially in line with the distribution of subjects within units that demonstrated Physics, followed by Biology as the highest representation. Therefore, the representation of cognitive expectations does not match the distribution of subjects. Therefore, we suggest considering the balanced distribution of tasks and activities in accordance with the distribution of subjects. Since problem-solving and problem-posing expectation types are rarely included in the textbooks, especially for inquiry activity and posing research questions in China [72], this finding suggests considering a fair inclusion of the related activities to the aforementioned types as science subjects in nature are more related to real-world contexts and are project-based with activated thinking skills.

The cognitive expectations were provided through several activities in the textbooks. The Chinese science textbooks' activities are distinguished in quantity and quality. A wide range of activities (e.g., Think and Discuss, Activity, Read Pictures, Experiment, Inquiry, and Exercise) leverage scientific thinking skills and active learning strategies. Particularly, chemistry put emphasis on carrying out experiments, and students are expected to employ their knowledge in the learning process [72]. The Egyptian science textbooks provide activities or exercises as cognitive expectations tasks whereas the procedure knowledge and representation levels are covered by a higher number of activities, unlike the conceptual knowledge and reasoning levels that are recognized with more exercises. The activities included in the textbook support students learning while conducting the experiments or observing the process of natural phenomena, so they are mostly used with procedure knowledge and representation levels; whilst the exercises basically appear at the end of the unit so they serve as assessment methods and were represented often with conceptual knowledge, asking students to memorize and recall the knowledge or clarify the reasons and speculation.

Regarding the learning goals, since the Egyptian science textbooks are goal-oriented, the number of intended goals outmatched the learning goals in the Chinese science textbooks. The vast majority of the goals aimed to help Egyptian students understand basic information and knowledge, e.g., distinguishing definitions and science concepts. Moreover, 25 % of the learning goals were stated to assist in understanding complex information, e.g., understanding the mechanism of a phenomenon. The considerable amount of exercises that represent cognitive expectations concern the conceptual knowledge level and are applied to assess the learning goals that focus on understanding basic and complex information. Therefore, we assume the relationship between learning goals and cognitive expectations to some extent. Similarly, we assume the relationship between the activities and exercises in the Chinese science textbooks which involve cognitive tasks, covering representation and procedure knowledge cognitive tasks and the learning goals of understanding the use of tools, routine procedures, and science processes. This emphasizes the diverse cognitive activities to allow students to discover, do experiments, read and observe, etc. to closely focus on the science processes and using tools and procedures. It also leverages the Nature of Science (NOS) as a critical element of scientific literacy that improves students' understanding of scientific ideas.

## 6 Conclusion, limitations and future work

This study sought to explore to what extent the ninth-grade science textbooks in Egypt and China are varied regarding their structure and content. An analysis of the school textbooks of the two countries was conducted. Chinese science textbooks are distinguished with the highest number of activities, figures, and lessons, while most of the activities in the Egyptian textbooks focused on Chemistry and Physics subjects. It is also seen that there is a dearth number of activities in Biology topics in Egyptian textbooks compared to Chinese ones in the same subject. The Egyptian science textbooks are goal-oriented focusing on presenting the learning goal at the beginning of the lessons. Furthermore, in terms of cognitive expectation, both Chinese and Egyptian textbooks highlighted representation, which is associated with obtaining as well as conveying information efficiently in scientific investigation. Egyptian textbooks emphasized conceptual knowledge much more than Chinese textbooks. In both countries' textbooks, the focus of cognitive requirements is the development of basic cognitive skills, such as understanding concepts, developing familiarity with the scientific inquiry procedure, and obtaining and conveying information from graphs and tables. Higher levels of cognitive skills, such as reasoning, problem-solving, and problem-posing, nevertheless, are not given enough priority in both countries' textbooks.

The Organization for Economic Co-operation and Development (OECD) contains the most developed economies in the world, which are also countries that have the most advanced science and technology. In OECD's PISA, scientific literacy is defined as “the ability to engage with science-related issues, and with the ideas of science, as a reflective citizen” [73]. Based on their definition, students with science literacy will be able to explain phenomena scientifically, evaluate and design scientific inquiry, and interpret data and evidence scientifically. More importantly, students are expected to apply scientific knowledge in the context of real-life situations. If this standard is used as the basis for evaluating the science textbooks in Egypt and China, it can be seen that both countries need to pay more attention to cultivating students' independent scientific inquiry skills as well as the connection of science knowledge to real-world settings.

The cognitive expectations promote procedure knowledge and reasoning expectation types in the Chinese science textbooks as prominent in Chemistry, while the prevalent cognitive tasks belong to Physics followed by Chemistry in Egypt but problem-solving and problem-posing expectation types are rarely included. A wide range of activities leverage the scientific thinking skills and active learning strategies in the Chinese science textbooks. The results of this research provide insights for improving the design quality of the preparatory stage textbooks, especially in science education. By identifying the main themes of the textbook's structure and content, this study can inform policymakers, educators, and curriculum developers in both Egypt and China (as well as worldwide) about the current state of science textbooks for the preparatory stage. The study also highlights the importance of considering cultural contextualization in textbook design.

Several limitations should be acknowledged in this study. First of all, since this study analyzed the structure and content of the school textbooks for the ninth grade in Egypt and China, the findings cannot be generalized to other grades or countries due to the differences in topics inclusions and cultures. Therefore, extended studies are encouraged to identify further features in textbook analysis between several countries and continents. Secondly, this study included only two Chinese science textbooks published by Zhejiang Education Press (ZEP version). Therefore, analyzing science textbooks published by other publishers could help to provide a comprehensive analysis and identify the differences and flows of textbooks designs as well as compare different periods. Thirdly, while we selected the science textbooks as a subject of the study, the analysis findings of other subjects can be varied because of the topic nature. Thus, future studies are advised to follow the analysis method applied in this study to different subjects in the preparatory stages, and investigate the effectiveness of textbooks on students' perceptions and readability [68]. Fourthly, we opted for quantitative analysis in this cross-country comparative study, focusing on structure and content analysis. We suggest implementing qualitative analysis and a more nuanced approach that considers the complex interplay of cultural, pedagogical, and contextual factors in textbook design and usage in the future. Besiddes, since this study's scope does not focus on assessing the readability and visual appeal of the textbook pages, we did not use the Page Surface Area (PSA) method in our analysis. According to the study findings and its limitations, we suggest formalizing international standards and criteria to analyze and evaluate the design quality of school textbooks, where these standards can guide teachers and students when using different forms of textbooks whether in classrooms or in blended learning environments. This emphasizes the importance of inclusive design of textbooks, calling for issuing accessible hard copies to serve students with disabilities.

## Data availability statement

Data will be available upon reasonable request.

## CRediT authorship contribution statement

**Ahmed Hosny Saleh Metwally:** Writing – review & editing, Writing – original draft, Visualization, Validation, Methodology, Formal analysis, Data curation, Conceptualization. **Ahmed Tlili:** Writing – review & editing, Validation, Resources, Project administration, Conceptualization. **Yiping Wang:** Writing – review & editing, Formal analysis, Data curation. **Zhimin Li:** Formal analysis, Data curation. **Jialu Zhao:** Formal analysis, Data curation. **Boulus Shehata:** Formal analysis, Data curation. **Dong Yang:** Writing – review & editing, Visualization, Resources. **Ronghuai Huang:** Supervision, Resources, Project administration, Conceptualization.

## Declaration of competing interest

The authors declare that they have no known competing financial interests or personal relationships that could have appeared to influence the work reported in this paper.

## Figures and Tables

**Fig. 1A:**
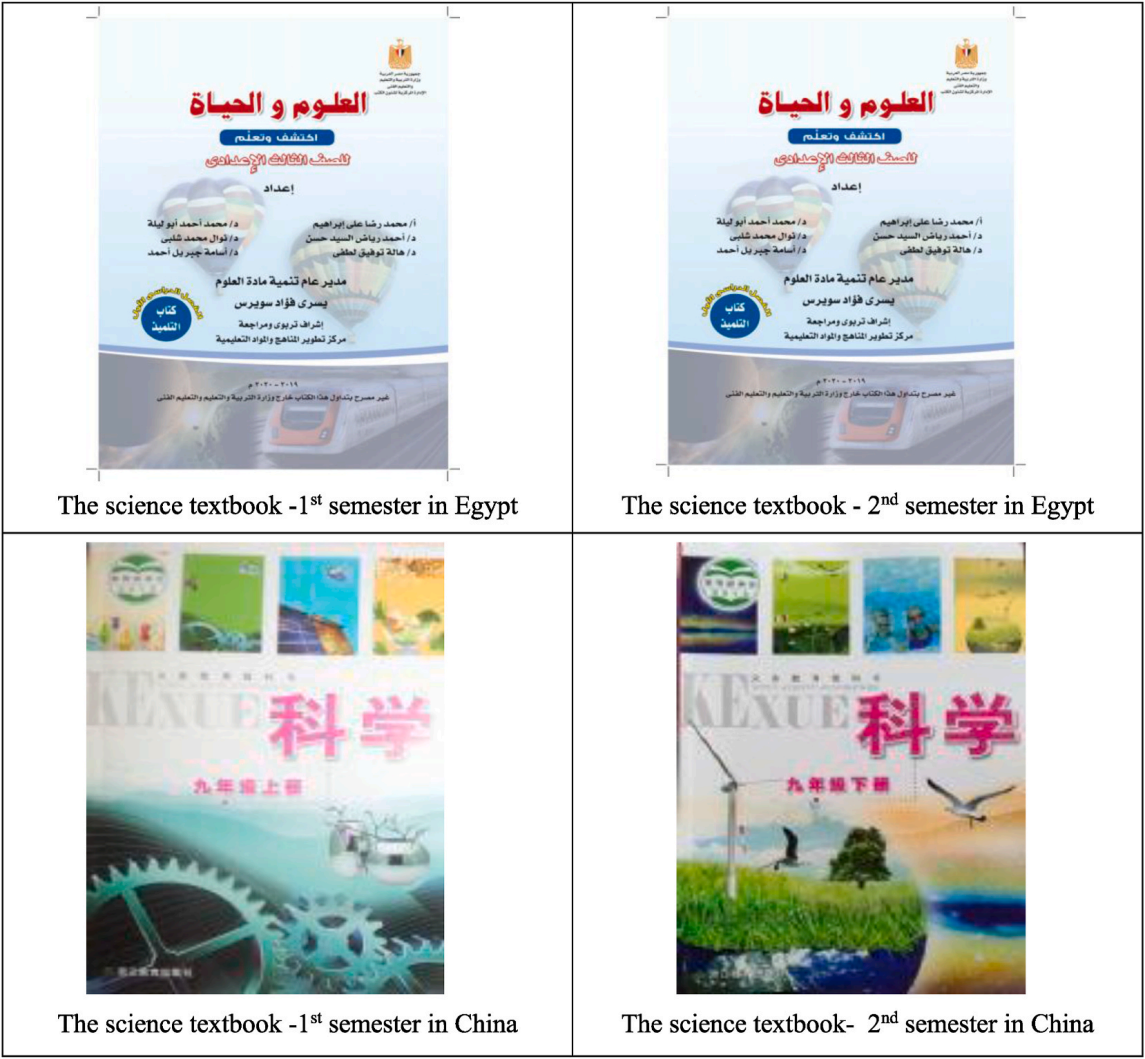
The cover page of the textbooks.
